# Exclusive enteral nutrition remodels the intestinal flora in patients with active Crohn's disease

**DOI:** 10.1186/s12876-022-02293-y

**Published:** 2022-04-30

**Authors:** Jingjing Jiang, Lu Chen, Yanfang Chen, Hong Chen

**Affiliations:** 1 School of Medicine, Southeast University, Nanjing, 210009 China; 2 Department of Gastroenterology, Zhongda Hospital, Southeast University, Nanjing, 210009 China; 3 Nanjing Medical University, Nanjing, China

**Keywords:** Crohn's disease, Exclusive enteral nutrition, 16S-rDNA, Intestinal flora, Short-chain fatty acid

## Abstract

**Background:**

Although there are many hypotheses, the pathogenesis of Crohn's disease (CD) is not completely clear so far. Exclusive enteral nutrition (EEN) is a routine measure in the treatment of active CD. We aimed at investigating the impact of EEN on patients with active CD from microbial metabolomics.

**Methods:**

16S-rDNA sequencing technology and gas chromatography–mass spectrometer analysis were employed to investigate the modification of the intestinal flora and fecal short-chain fatty acid (SCFA) during the EEN.

**Results:**

Seven patients with CD, who conducted EEN, were followed up successfully in the present study. The 8-week EEN resulted in a remission of the condition of subjects with active CD, as revealed by a significant decrease in erythrocyte sedimentation rate (ESR) (*P* = 0.018), C-reactive protein (CRP) (*P* = 0.028), and Crohn’s disease activity index (CDAI) (*P* = 0.018). The nutrition of the subjects was improved after an 8-week treatment course with EEN, which was associated with an increase in body mess index (BMI) (*P* = 0.018) and serum albumin (ALB) (*P* = 0.018) levels. Furthermore, our investigations revealed a significantly increased abundance of Firmicutes paralleled by decreased levels of Proteobacteria. With respect to the genus, five species of bacteria including Ruminococcus (*P* = 0.01), Lachnospiraceae (*P* = 0.02), Anaerotruncus (*P* = 0.04), Flavonifractor (*P* = 0.04), and Novosphingobium (*P* = 0.05) showed significantly increased abundance. This was accompanied by relative changes in fecal short-chain fatty acids levels. Moreover, we successfully constructed a stable model by combining these five significantly different genera to predict the therapeutic effect of EEN on patients with CD (AUC = 0.9598).

**Conclusions:**

The findings indicated that EEN can alleviate the condition and the nutrition of patients with active CD by regulating the intestinal flora and influencing the expression level of fecal short-chain fatty acids.

**Supplementary Information:**

The online version contains supplementary material available at 10.1186/s12876-022-02293-y.

## Introduction

Crohn's disease (CD) is an inflammatory bowel disease (IBD), characterized by recurrent illness. The incidence and prevalence of CD have risen during the last decades, but the specific etiology and pathogenesis are still unclear [1]. The clinical manifestations of CD often occur as the alternation of disease remission and relapse [2]. The current clinical treatment of CD is mainly based on drug-based induction therapy such as glucocorticoids, immunosuppressant drugs, and biological agents.

Existing studies have confirmed that the application of exclusive enteral nutrition (EEN) had a significant effect on the healing of intestinal mucous membrane and recovery of intestinal function in patients with CD [3]. Early implementation of EEN has a higher therapeutic value for the long-term prognosis of CD [4]. According to domestic and foreign guidelines and consensus [5, 6], EEN is one of the preferences for patients with CD, severe cases included, and is the first-line induction therapy for children and adolescents. Based on available single-center cohort studies, some patients with CD can achieve a remission rate of up to 80% by applying EEN [6–9]. The therapeutic effect of EEN on patients with CD is not only inducing the remission of inflammation but also offering nutritional support [10, 11]. And EEN is also a relatively economic treatment. Plenty of patients cannot tolerate corticosteroid and immunosuppressive therapy [12]. Moreover, some cannot afford expensive biologics. Under such circumstances, EEN may even be the only effective treatment option.

Intestinal flora plays an important role in mucosal immune regulation. Intestinal symbiotic bacteria can inhibit the growth of pathogenic bacteria and maintain intestinal homeostasis [13]. Short-chain fatty acid (SCFA) represents metabolites produced by the intestinal flora and might occupy an important position in the occurrence and development of CD [14–16]. According to Ceylan Tanes et al. [17], EEN or other special dietary might change the structure of specific metabolites by affecting the microbiome of healthy volunteers, thus affecting the inflammatory process. Whether this phenomenon is the same in patients with CD needs further testing. The present study aimed to investigate the changes of the intestinal flora and fecal SCFAs level after the EEN in patients with CD.

## Methods

### Study subjects

The patients who were diagnosed with Crohn's disease in the Department of Gastroenterology, Zhongda Hospital Southeast University from October 2018 to December 2019 were included in the study. The selection of subjects was based on the Crohn's disease diagnostic criteria and Crohn's disease activity index (CDAI) in the ‘2018 Consensus Opinions on the Diagnosis and Treatment of Inflammatory Bowel Disease' released by the Inflammatory Bowel Disease Group of the Chinese Medical Association Gastroenterology Branch [5]. The Montreal classification was applied to type the subjects [18].

The inclusion criteria of our subjects were as follows: (1) patients with active Crohn's disease who had already been treated with infliximab (Janssen-Cilag AG, USA) for more than 12 weeks but responded poorly; (2) age ≤ 75 years old; (3) no administration of antibiotics or probiotics, for at least 1 month before entering the study; No additional treatment regimens (such as glucocorticoids, biological agents or surgery) were newly employed during the period of EEN; CDAI score ≥ 150 points, i.e. in the active phase; All participants approved the informed consent before conducting experiments. Infection before or during the study as well as incapability of taking oral or intestinal food and suffering from dairy allergy represented the exclusion criteria.

The enteral nutrition used was Enteral Nutritional Powder (TP) (ENSURE, Abbott Laboratories B.V., USA). This represents a compound preparation that contains protein, fat, carbohydrates, vitamins and minerals. 55.8 g of powder can be mixed with 200 ml of water to prepare a 250 ml dose per serving. It provides 1.06 kcal energy per milliliter, with a calorie distribution of 54% carbohydrate, 31.8% fat, 14.2% protein, and a variety of vitamins and minerals. This compound can be delivered either orally or tube-fed. All participants lived in Nanjing, Jiangsu Province, or in Nanjing's surrounding counties and cities for a long time with comparable lifestyles, climate environments and dietary habits. Our study was approved by the Clinical Research Ethics Committee of Zhongda Hospital Southeast University (Approval Number: 2020ZDSYLL129-P01).

### Sample collection

Blood samples were collected before and after 8-week EEN to determine the inflammation index, namely erythrocyte sedimentation rate (ESR) and C-reactive protein (CRP). The fresh stool samples were taken and loaded into a sterile dry stool kit at baseline time and after 8 weeks of EEN. The approximate weight of the stool specimen was about 3.0 g. Upon collection and labeling, samples were quickly frozen in liquid nitrogen and stored in the refrigerator at minus 80 degrees centigrade.

### Sample preparation and 16S-rDNA sequencing

The E.Z.N.A.®Stool DNA Kit for extraction of fecal bacterial DNA was used according to the manufacturer's instructions. During the whole process of DNA extraction, ultra-pure water was used to avoid false positive PCR results. The PCR products were purified by the AMPure XT beads (Beckman Coulter Genomics, Danvers, MA, USA) and quantified by Qubit (Invitrogen, USA). Before sequencing, the size and quantity of the amplicon pool were evaluated on the Agilent 2100 Bioanalyzer (Agilent, USA) and Illumina (Kapa Biosciences, Woburn, MA, USA) library quantification kits. Finally, we sequenced the libraries on the NovaSeq PE250 platform.

### Analysis of fecal short-chain fatty acids

Twenty milligrams of each fecal sample were drawn out and mixed with isobutanol solution in a volume ratio of 1:9 before grinding and centrifugation. Sodium hydroxide and chloroform were added to the supernatant before another centrifugation round. This supernatant was retrieved and subjected to derivatized with chloroformate. Finally, the ultimate supernatant was extracted with normal hexane to detect the level of SCFAs by GC–MS (Agilent, USA).

### Statistical analysis

The sample sequencing in this study was performed on the Illumina NovaSeq platform provided by Lc-bio. All data calculations were performed using QIIME2. Data analysis and preparation of charts were performed using the R software package (v3.5.2) and online cloud platform (Lc-bio) get on. To accurately compare the similarities and differences in the composition of intestinal flora before and after treatment, we involved Multidimensional scaling analysis (NMDS analysis), Principal coordinates analysis (PCoA analysis) and Analysis of similarities (Anosim analysis) based on the OTUs of the sample. NMDS analysis under Weighted-UniFrac distance on the OTU levels of all fecal samples was conducted. The data collection and analysis of SCFAs involved in this study were performed using Mass Hunter software (Version B.08.00, Agilent, USA).

## Results

### Study subjects

**Fig. 1:**
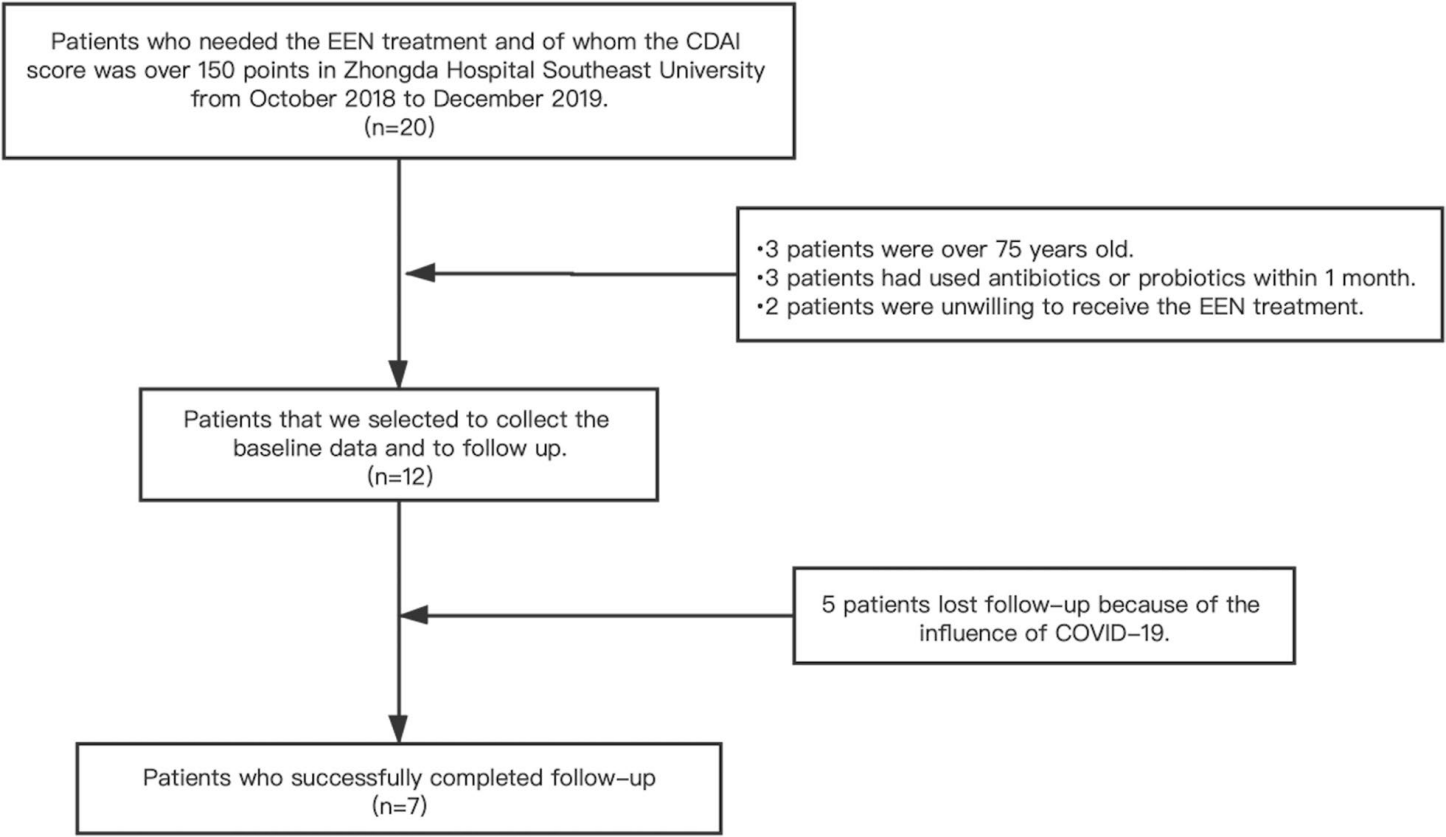
Flow chart depicting the procedure of subjects' registration is presented

Twenty patients with active CD (CDAI > 150) who were going to be conducted EEN were initially entered into the study. Eight patients were excluded because of the eligibility criteria: three of them were > 75 years old; three had used antibiotics or probiotics within 1 month and two patients were unwilling to receive the EEN for a long time. The remaining 12 patients were subjected to a collection of baseline data and follow-up. Due to the COVID-19 pandemic, five patients were missed in the follow-up analysis. As a result, seven subjects successfully completed the follow-up analysis (Fig. 1).

Seven patients with active CD (CDAI > 150) were selected for our study (Fig. 1). We applied EEN for 8 weeks to the 7 subjects, using ENSURE as the enteral nutrition. The cohort was included 5 males (72%) and 2 females (28%) with a median age of 25.0 years. According to the location of CD, 43% of the subjects featured ileal disease (L1), 14% displayed colonic disease (L2), while 43% showed ileocolonic Crohn's disease (L3) (Table 1). The median ESR before EEN (Group Prior) was 23 mm/h, while the median hypersensitive CRP was 7.67 mg/L. After 8 weeks of EEN (Group Post) revealed a decreased median ESR (4 mm/h) and CRP (0.86 mg/L). Wilcoxon rank-sum test was used to compare the ESR and CRP in the two groups. The result suggested that the level of ESR and CRP significantly decreased after the 8-week EEN (*P* = 0.018 and *P* = 0.028 respectively). At the same time, a significant decrease in CDAI after EEN (*P* = 0.018) was observed, suggesting that EEN was effective in treating patients with active CD in this study. In addition, various baseline parameters of the subjects related to our study, including weight, height, body mess index (BMI), and serum albumin (ALB) were documented (Table 1). Our investigations revealed increased BMI and ALB after the EEN, suggesting that the nutritional status of subjects was obviously improved (*P* = 0.018).

**Table 1:** Clinical Characteristics of 7 patients with CD (Prior EEN/Post EEN)

ID	Sex	Age	Montreal	Previous relevant medications	BMI (Pr*/Po*)	CDAI (Pr/Po)	ESR (mm/h) (Pr/Po)	CRP (mg/L) (Pr/Po)	ALB (g/L) (Pr/Po)
01	M	16	A1L3B1p	5-ASA/IFX	12.80/14.87	329.9/128.1	74/11	79.6/0.86	29.8/42.1
02	F	25	A2L2B1	Pred/5-ASA/IFX	17.19/17.97	250.87/107.89	35/2	7.67/0.86	33.1/44.6
03	F	31	A2L3B3p	5-ASA/IFX	16.41/18.75	257.44/118.24	23/18	7.38/0.86	35.3/38.3
04	M	23	A2L1B2	AZA/IFX	16.98/17.63	249.37/129.2	3/2	1.2/1.58	48.8/50.5
05	M	15	A1L1B1p	IFX	14.80/17.36	293.71/144.47	8/4	32.47/2.01	32.1/39.6
06	M	51	A3L3B3	5-ASA/IFX	20.43/20.70	385.8/108.47	5/4	16.4/0.78	24.7/32.8
07	M	34	A2L1B1	AZA/IFX	21.51/21.97	212.26/86.42	32.95/4	5.85/0.78	38.5/40.7

*CD* Crohn’s disease, *M* male, *F* female, *Pr* Prior, *Po* Post, *BMI* body mess index, *5-ASA* 5-aminosalicylic acid, *IFX* infliximab, *Pred* Prednisone, *AZA* Azathioprine, *CDAI* Crohn's disease activity index, *ESR* erythrocyte sedimentation rate, *CRP* C-reactive protein, *ALB *serum albumin

*Pr represents the baseline collection time point and Po represents 8 weeks of EEN

### Effective sequencing sequence statistics

Double-ended splicing, quality controlling, and mosaic filtering of the original off-machine data, were followed by high-quality data statistics. The effective sequencing sequence statistics are presented in Table S1 (Additional file 1).

**Fig. 2:**
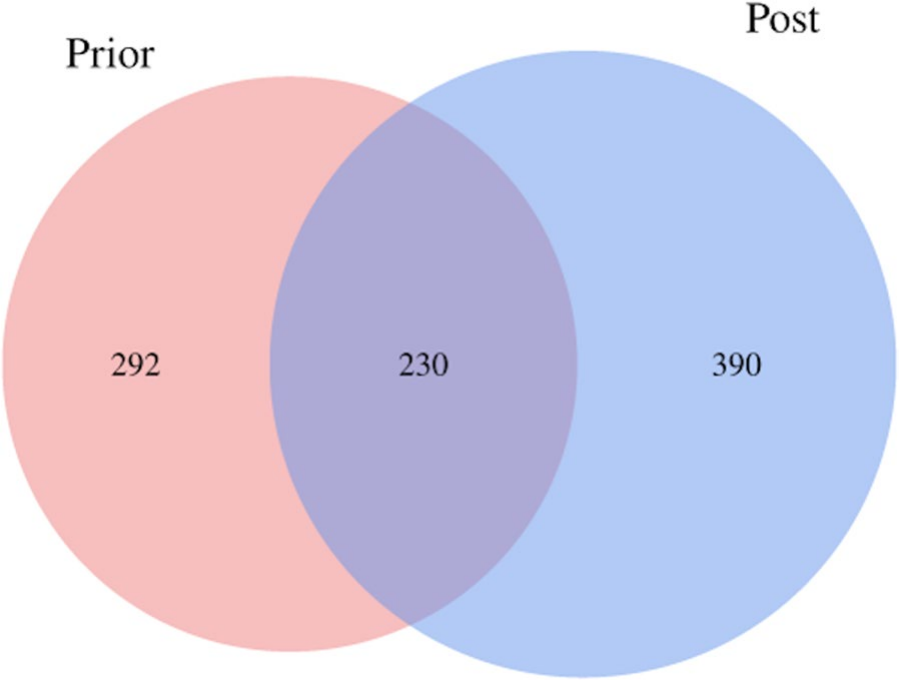
Venn diagram of the operational taxonomic units (OTUs) in group prior (red), in group post (green), and the overlap OTUs between the two groups. The number of OTUs of the samples before the EEN is 292, and that of the samples after 8-week EEN is 390. The number of the overlap OTUs is 230

The statistical analysis of the abundance indicates that the two groups have 230 identical bacteria in their respective stool sample flora as presented in the Venn diagram (Fig. 2). However, the two groups have their own relatively unique bacteria as well. Group Post has a higher number of unique bacteria than Group Prior (390 vs. 292). This indicates that the fecal flora species of patients with CD increased after the 8-week of EEN.

### Diversity analysis of the intestinal flora after the EEN

**Fig. 3:**
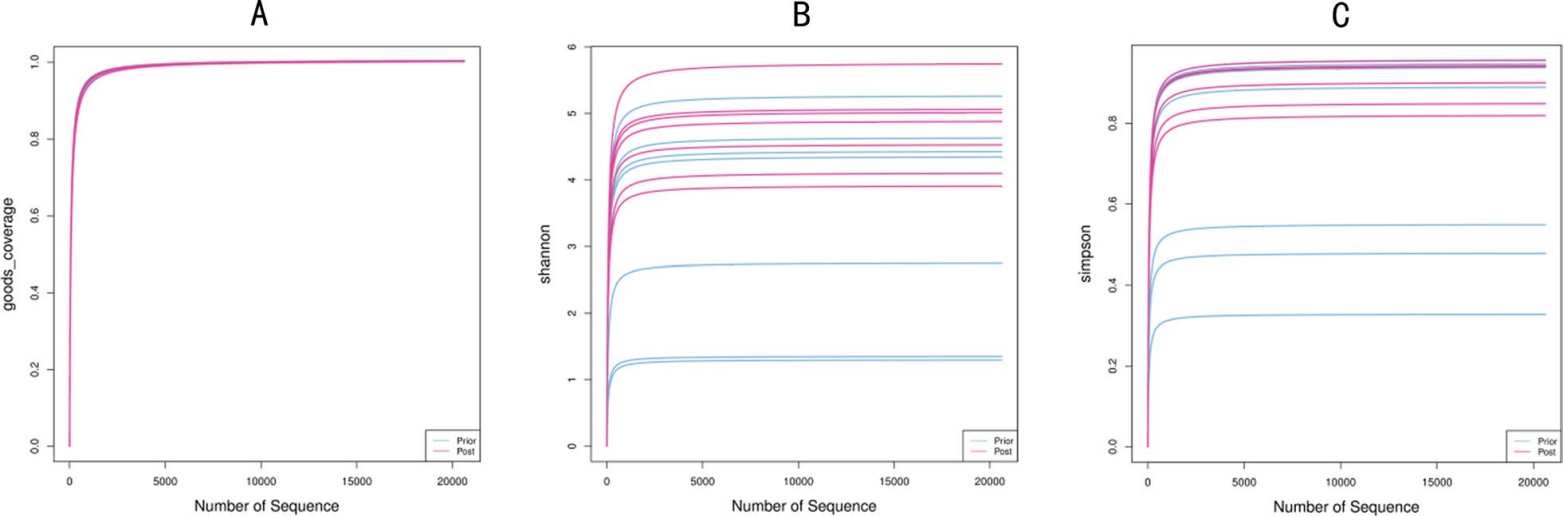
**a** Rarefaction curve of the alpha diversity in each sample is presented. **b**, **c** Alpha diversity analyses, including Shannon index and Simpson index, are shown. Shannon index represents the species diversity of the sample. Simpson index reflects the species richness and evenness of the sample

**Fig. 4:**
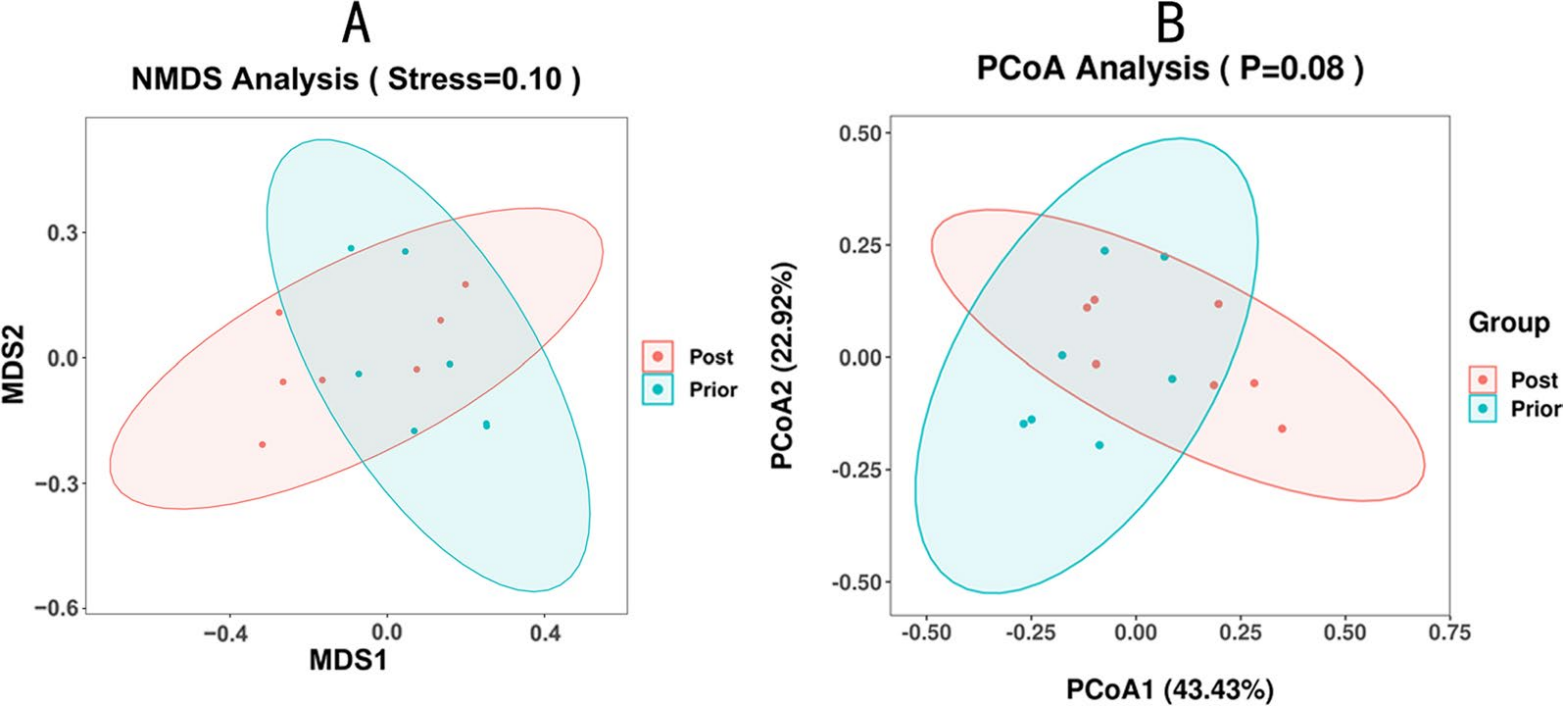
**a** NMDS analysis under weighted-Unifrac distance on the OTUs of Group Prior (blue) and Group Post (red) are presented. **b** PCoA under weighted-Unifrac distance on the OTUs of Group Prior (blue) and Group Post (red) are shown

The Goods-coverage curve reflects that sample feature coverage is infinitely close to 100%. This suggests that the sequencing results may represent the true condition of these 14 samples (Fig. 3a). Upon applying Shannon and Simpson indexes to evaluate the alpha diversity of the intestinal flora of the two groups, we observed that the diversity of the Group Post is higher than that of Group Prior at the same sequencing depth. This indicates an improvement in the abundance and diversity of the intestinal flora of patients with CD after EEN (Fig. 3b, c). The alpha diversity dilution curve of this study gradually stabilized as the amount of sequencing data increased, indicating that the amount of sample sequencing has been saturated. The analysis of alpha diversity revealed that although both groups have high flora abundance and diversity, Group Post shows even higher levels. Our investigations indicate a certain degree of clustering of OTUs between the samples (Fig. 4) with obvious separation of the intestinal flora of Group Prior and Group Post. This means that the two groups of flora have low similarity which suggests that the composition of intestinal flora, (i.e. the intestinal microecology of patients with CD after EEN), has changed to a certain extent. Furthermore, the value of Stress (i.e. Stress = 0.1), namely the pressure coefficient, indicates the high reliability of the NMDS analysis results (Fig. 4a).

These findings were substantiated by PCoA analysis of the samples under Weighted-UniFrac distance. We found that both PCoA1 (43.43%) and PCoA2 (22.92%), account for 66.35% of the sample difference. Figure 4b shows a certain distance between the two groups in the first and the second principal coordinates. This infers a potential difference between the microbial composition of Group Prior and Group Post, despite no significant values (*P* = 0.08) (Fig. 4b). Anosim analysis compares the difference between the groups with the difference within the groups. The calculated R value of 0.017104, indicates that the difference between the groups is greater than the difference within the same group. This suggests that the grouping of our study is significant.

Moreover, the beta analysis of the samples revealed that the EEN renders an influence on the composition of the intestinal flora of the active CD patients.

### Analysis of the composition and abundance of bacterial species

**Fig. 5:**
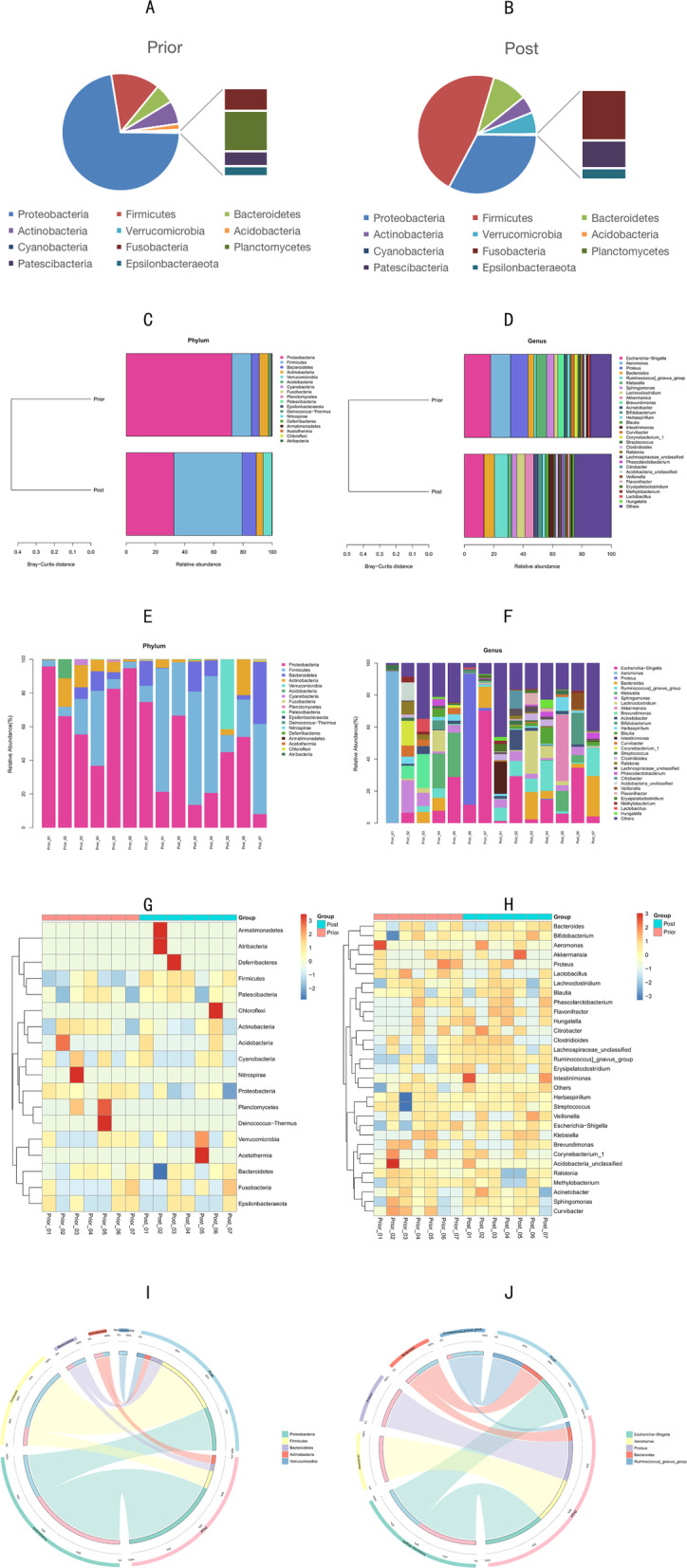
**a**, **b** Pie charts of the abundance ratio of the bacteria in terms of phylum level in Group Prior and Group Post are depicted. **c**, **d** Cluster analysis of the relative abundances in Group Prior and Group Post with the Bray–Curtis distance are shown. **e**, **f** Bar charts of the relative abundances among all samples with the Bray–Curtis distance are presented. **g**, **h** The relationship between the 14 samples and the abundance of the top 30 phyla and genera is presented by the two heatmaps in which the Z value was used to normalize the expression abundance of the same bacteria. The gradient from blue to red reflects the change in abundance from low to high. **i**, **j** Circos charts show the correspondence between samples and species from phylum and genus level, reflecting the composition ratio of dominant species in Group Prior and Group Post, and reflecting the distribution ratio of each dominant species among the two groups are depicted

In terms of phylum level, the intestinal microbiota of the patients with CD before EEN comprises mainly of *Proteobacteria*, *Firmicutes*, *Bacteroides*, *Actinobacteria* and *Acidobacteria*. At the same time, the intestinal microbiota of the fecal samples of Group Post is mainly composed of *Proteobacteria*, *Firmicutes*, *Bacteroides*, *Actinobacteria* and *Verrucomicrobia*. Besides, in terms of genus level, Group Prior is dominated by *Escherichia-Shigella*, *Aeromonas*, and *Proteus.* These account for 17.85%, 13.5%, and 11.92% respectively of the total flora. Additionally, the Group Post is dominated by *Escherichia-Shigella*, *Ruminococcus*, and *Bacteroides*, respectively accounting for 13.17%, 9.24% and 7.19% of the entire flora composition (Fig. 5).

### Analysis of the species differences between group prior and group post

**Fig. 6:**
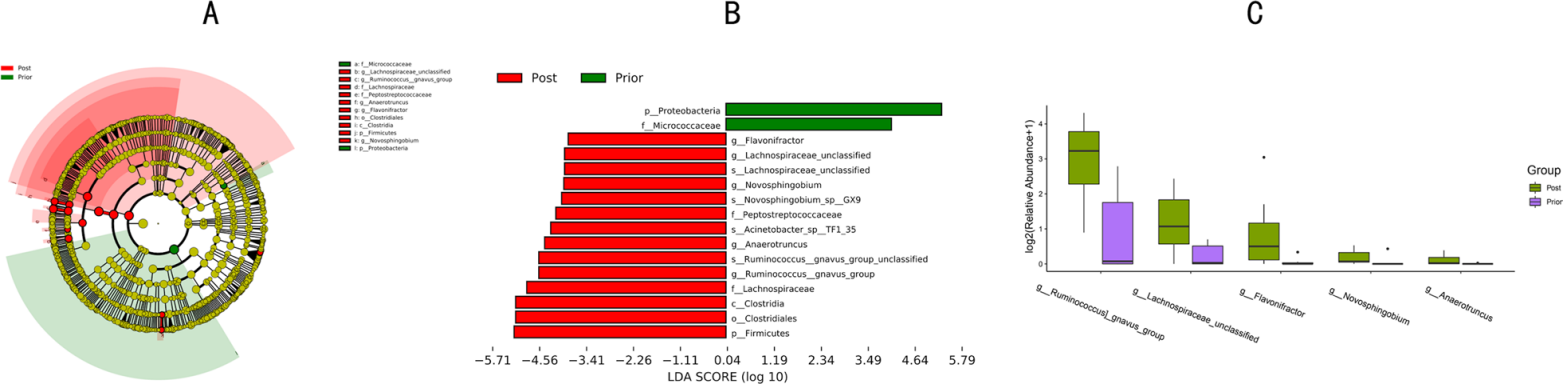
**a**, **b** LEfSe analysis reveals significant differences in abundance between the two groups. The green node indicates higher species abundancy in group A; the red indicates higher species abundancy in group B. Yellow node reveals no significant difference between the two groups. **c** The barplot analysis shows the five genera with significant differences between Group Prior and Group Post

By employing LDA Effect Size analysis (LEfSe difference analysis) of the subjects' samples, we identified and compared the species with significant differences between Group Prior and Group Post. Figure 6a and b indicate that at the phylum level, *Proteus* has a higher abundance in Group Prior, while *Firmicutes* has a higher abundance in Group Post. In other words, the 8-week EEN was associated with an increased proportion of *Firmicutes* and decreased proportion of *Proteus* in the intestinal flora. In addition, the EEN was corroborated with a higher abundance of *Lachnospiraceae*, *Ruminococcus*, *Anaerotruncus*, *Flavonifractor*, and *Novosphingobium* (significant differences between Group Prior and Group Post, with* P* values of 0.01, 0.02, 0.04, 0.04 and 0.05 respectively). For a better view, we displayed the differences in the fecal flora as a bar plot. (Fig. 6c).

### Prediction model based on significantly different intestinal flora

**Fig. 7:**
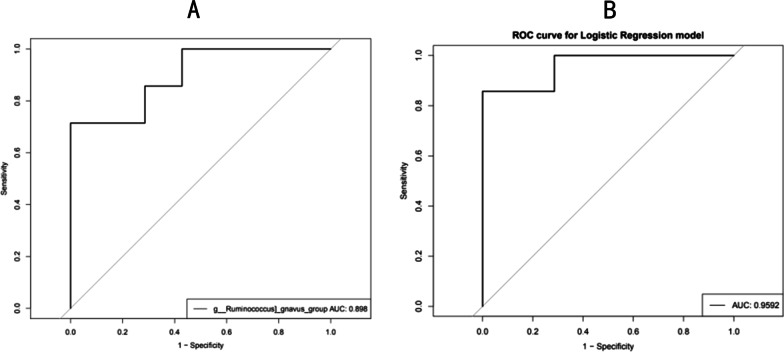
**a** ROC curve of *Ruminococcus*. **b** ROC curve that combines the five genus bacterium, *Ruminococcus*, *Lachnospiraceae*, *Flavonifractor*, *Novosphingobium* and *Anaerotruncus*

Based on the LDA value of the LEfSe analysis, we selected *Lachnospiraceae*, *Ruminococcus*, *Anaerotruncus*, *Flavonifractor*, and *Novosphingobium* as the candidate bacterial markers, and then performed ROC analysis to explore the predictive ability of the intestinal flora before and after treatment of patients with CD. The ROC curve of *Ruminococcus* suggested this genus as the most reliable when distinguishing patients with CD before and after EEN (AUC = 0.898) (Fig. 7a).

The ROC analysis of five bacteria genera easily allows distinguishing between Group Prior and Group Post (AUC = 0.9592) (Fig. 7b). This helped us to construct a stable and statistically significant optimal prediction model upon a combination of the five bacteria genera *Lachnospiraceae*, *Ruminococcus*, *Anaerotruncus*, *Flavonifractor* and *Novosphingobium*.

### Analysis of fecal short-chain fatty acids

**Fig. 8:**
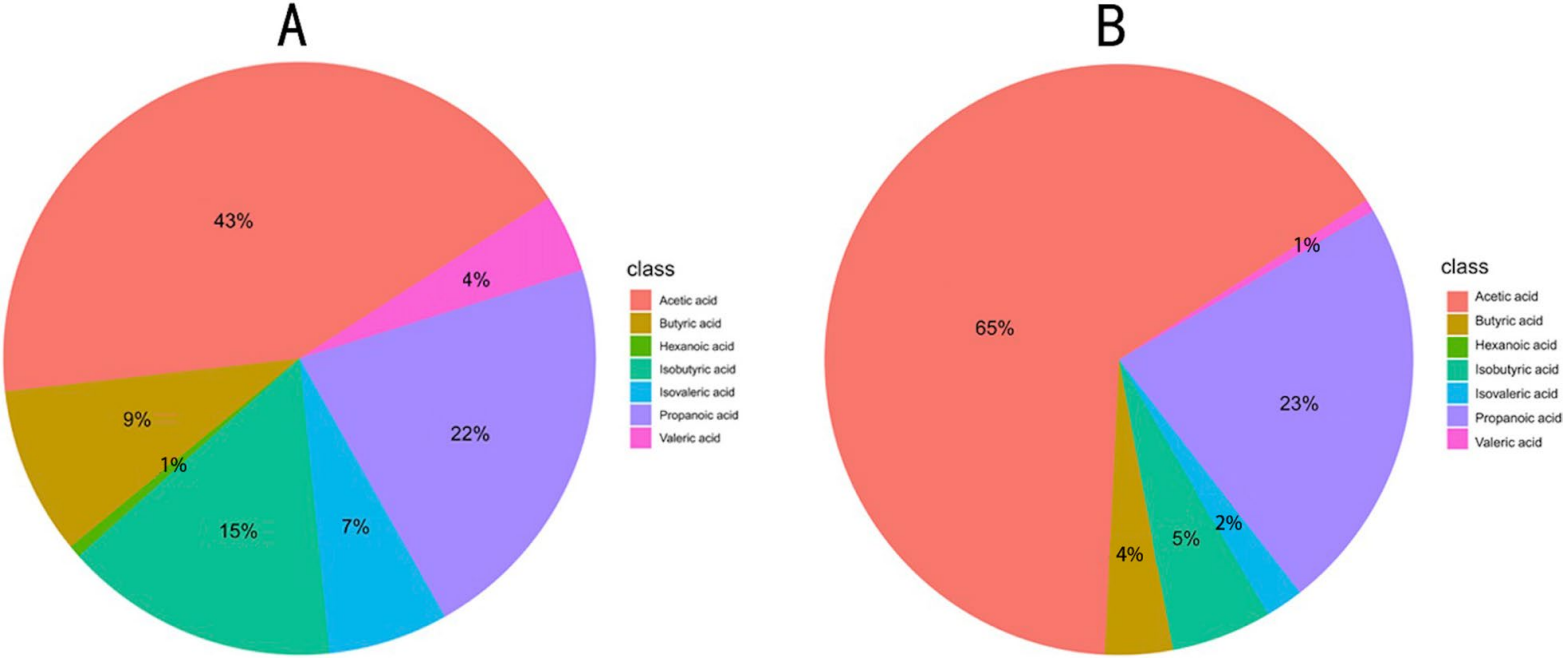
**a**  Pie chart of the proportion of each SCFA in Group Prior.  **b**  Pie chart of the proportion of each SCFA in Group Post

We measured the fecal SCFAs levels of the two groups by GC–MS in order to reveal the potential changes before and after the EEN. In our research, we found that the most abundant SCFA in all seven active CD patients' stool was acetic acid (43%), propanoic acid (22%), butyric acid (9%), isobutyric acid (15%) and isovaleric acid (7%), while valeric acid and caproic acid accounted for only a small ratio of the overall (4% and 1%). After EEN, the composition of SCFAs in the patients’ feces has changed. Here acetic acid had the highest proportion of all SCFAs (65%) (Fig. 8a, b).

**Fig. 9:**
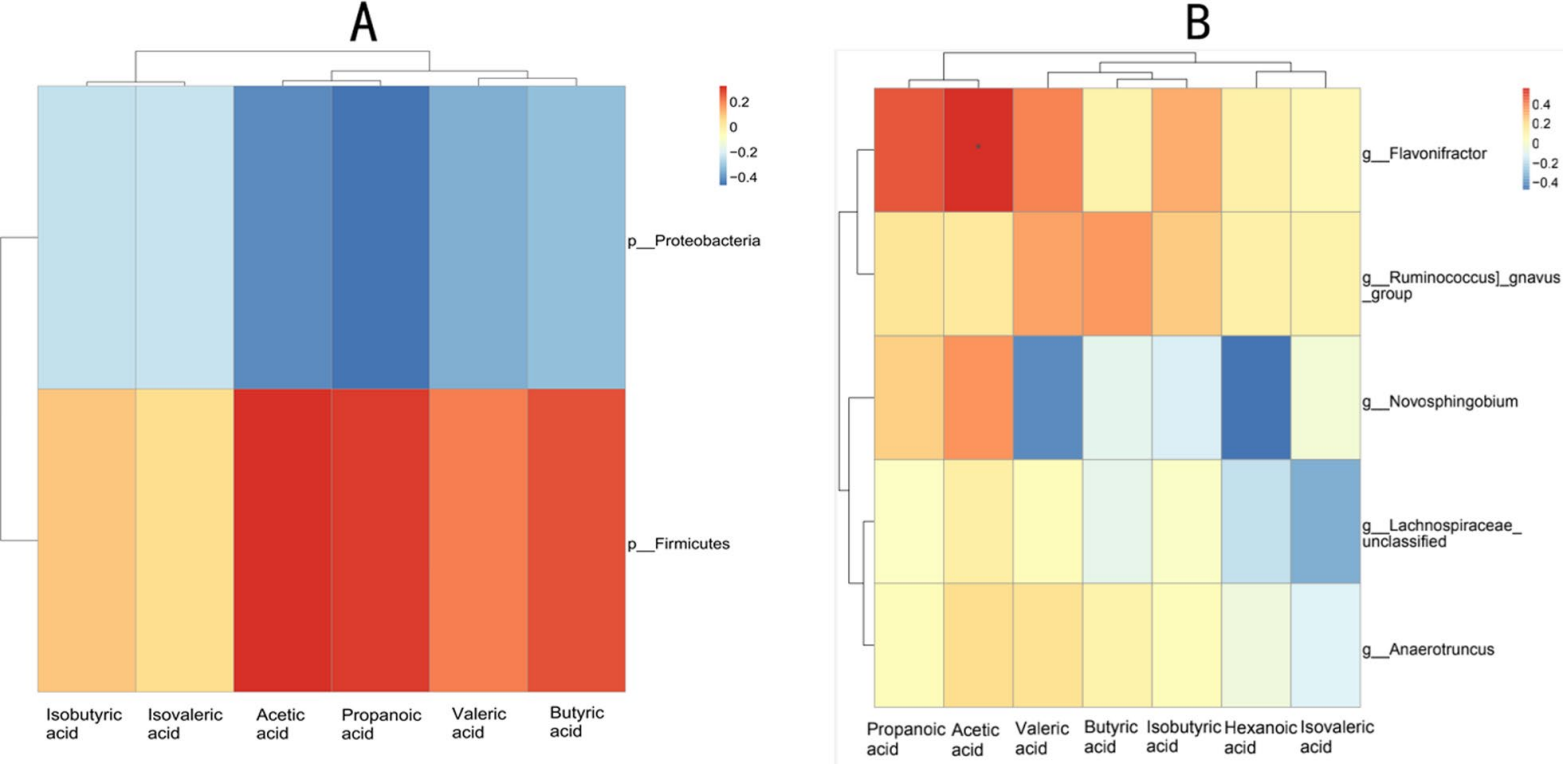
**a** Spearman correlation heatmap of the relationship between the two significantly different bacterial phyla and the SCFAs. **b** Spearman correlation heatmap of the relationship between the five significantly different bacterial genera and the SCFAs

We also combined the abundance of intestinal flora with the level of SCFAs to conduct a Spearman correlation analysis. Here we investigated and represented as a heatmap the correlation between intestinal flora and SCFAs level. Spearman analysis revealed a positive correlation between *Firmicutes* abundance and the level of fecal SCFAs, especially acetic acid, propanoic acid, butyric acid and valeric acid. Additionally, the analysis indicated a negative correlation between the abundance of *Proteobacteria*, and the predominant acetic acid and propanoic acid (Fig. 9a). In addition, our data also showed that, in terms of the genus, *Flavonifractor* and *Ruminococcus* are positively correlated with all fecal SCFAs and especially with the levels of acetic acid, propanoic acid, and valeric acid. On the other hand, the levels of butyric acid, isobutyric acid and valeric acid are negatively correlated with *Novosphingobium* (Fig. 9b). Altogether, our results revealed a significant correlation between changes in fecal SCFAs levels and the abundance of intestinal flora.

## Discussion

In the present study, we show that the active condition of all 7 subjects involved, had gained obvious remission after the EEN. This was reflected in the significant reduction of the CDAI, ESR and CRP. Furthermore, our investigations revealed that the subjects' nutrition has been significantly improved after the 8 weeks of treatment. The findings show a change in the composition and the abundance of intestinal flora as well as of SCFAs metabolites' levels after the EEN. In addition, our study delineates a correlation between the changes in the bacterial community and SCFAs. As hypothesized earlier, all of these findings indicate that the EEN may relieve the active state of patients with CD by regulating the composition of the intestinal flora and improving the levels of SCFAs.

Previous studies reported that the intestinal flora of healthy people is mainly composed of several major species such as *Firmicutes* and *Bacteroides* [19]. At the same time, the diversity of the intestinal flora of patients with CD is significantly reduced and sometimes, the core flora may further decrease or even disappear. On the other hand, *Enterobacterium* might significantly increase on the intestinal mucous membrane of these patients [20]. Extensive studies showed that the disorder of the intestinal flora is closely associated with inflammatory bowel disease, including CD. In other words, the intestinal flora of patients with CD is substantially imbalanced [21]. According to a study by Ignacio Catalan-Serra et al. [22], the number of intestinal flora in patients with CD is lower than that documented for healthy individuals. Besides, the changes reflect a decrease in the number of *Firmicutes*, *Bacteroides* and other flora, and the overexpression of Enterobacter. Furthermore, Zhang et al. [13] found that the number of opportunistic pathogens, mainly represented by *Escherichia coli*, in the stool of the patients with active CD was higher than that of patients whose condition was in remission.

Our analysis of fecal bacterium abundance revealed that patients with active CD show an increase of fecal flora composition after EEN compared with that before the treatment. Particularly, the abundance of *Firmicutes* significantly increased and became the dominant bacteria after the 8-week EEN. At the same time, the abundance of *Proteobacteria* significantly decreased. The analysis at the genus level revealed that the abundance of *Ruminococcus*, *Novosphingobium*, *Lachnospiraceae*, *Anaerotruncus,* and *Flavonifractor* significantly increased after treatment. Matthew et al. [23] found that *Ruminococcus* can synthesize and secrete a complex glucomannan polysaccharide with a rhamnose backbone and glucose side chains, thereby it can effectively induce dendritic cells to secrete inflammatory cytokine (TNF-α). In addition, *Ruminococcus* can utilize mucin as a carbon source to directly destroy intestinal barrier function. These both have been associated with the occurrence and development of CD inflammation [23]. Indeed, the findings of our study prove the hypothesis that EEN alleviates the condition of patients with CD by adapting or modulating the structure and composition of the intestinal flora [24].

The analysis of fecal SCFAs levels demonstrated that the level of short-chain fatty acids also changed after EEN. Particularly an increased amount of acetic acid was documented. The Spearman correlation analysis involving two bacterial phyla and 5 bacterial genera indicated significant differences before and after EEN with respect to the fecal SCFAs. Interestingly, the levels of acetic and propanoic acids were positively correlated with the abundance of *Firmicutes*, but negatively correlated with the abundance of *Proteus* phylum. In terms of the genus, *Flavonifractor* and *Ruminococcus* were positively correlated with all SCFAs in the fecal samples. It means that the effectiveness of EEN is directly proportional to the changes in SCFAs levels. It does suggest that the simultaneous disorder of intestinal flora and the imbalance of SCFAs metabolites represent regulatory factors that relate to the condition of CD. In this way, EEN may alleviate the condition of these patients by ameliorating intestinal flora and metabolism.

Notably, we also selected the five bacterial genera (i.e. *Ruminococcus*, *Lachnospiraceae*, *Anaerotruncus*, *Flavonifractor* and *Novosphingobium*) that had significant differences in the changes of abundance during the EEN of CD patients. ROC analysis was employed to distinguish the status before and after treatment based on intestinal flora populations. Our investigations demonstrate that *Ruminococcus* abundance provides essential information on the state of patients with CD before and after EEN. Furthermore, the combined prediction model of these 5 species of bacteria allows for more accurate prediction.

In conclusion, our study firstly delineated the changes in the composition and abundance of the intestinal flora of patients with active CD after EEN, as well as the changes in the level of their metabolites SCFAs in feces. Secondly, our investigations revealed a direct correlation between the level of SCFAs and changes in bacterial abundance, suggesting a synergistic effect of the two which might be related to the EEN mechanism. Thirdly, the study delineated that levels of acetic acid, propanoic acid, and butyric acid are related to changes in the intestinal flora. As SCFAs members, they may be used as new sensitive indicators to help in better understanding the onset and the development of CD. The last but not least, we successfully designed a reliable and stable prediction model upon the combination of 5 specific bacteria. Altogether, our study demonstrates that the change in the intestinal flora represents a positive and effective basis for CD treatments.

The present study still has some limitations. Firstly, only 7 subjects were successfully followed up. Usually, after patients with CD respond poorly to infliximab (IFX), the processing scheme is increasing the dose of IFX or shortening the interval, for the patients who don’t have high anti-IFX antibodies, or switching to another biological agent. When this study went on, all of the biologics for CD had not been included in the Chinese basic medication insurance, and the patients had to pay for biologics on their own. That is a very large expense and a very important interference factor in making a medication schedule. Just for this reason, the 7 subjects in this study were unable to burden more expensive biologics. So, we selected to conduct EEN only to them. Secondly, the 16S-rDNA prediction function is rather limited if it is compared with the prediction function of metagenomics. Metagenomics can further clarify and delineate the function of flora, revealing the possible mechanism of intestinal flora and SCFAs affecting CD as well as the mechanism behind the EEN. This may further bring insight into Crohn's disease, an illness with unclear pathogenesis to date, and guide the discovery of new therapeutic strategies for CD.

## Supplementary Information

**Additional file 1: Table S1.** The effective sequencing sequence statistics of the Fecal sample.

## Data Availability

The data used and analyzed during the present study are available from the corresponding authors upon reasonable request. The data are not publicly available due to privacy restrictions.

## Abbreviations

CD: Crohn's disease
EEN: Exclusive enteral nutrition
IBD: Inflammatory bowel disease
SCFA: Short-chain fatty acid
GC–MS: Gas chromatography–mass spectrometer technology
5-ASA: 5-Aminosalicylic acid
IFX: Infliximab
Pred: Prednisone
AZA: Azathioprine
CDAI: Crohn's disease activity index
ESR: Erythrocyte sedimentation rate
CRP: C-reactive protein
BMI: Body mess index
ALB: Serum albumin
OUT: Operational Taxonomic Unit
NMDS: Multidimensional scaling analysis
PCoA: Principal coordinates analysis
LEfSe: LDA effect size analysis
