# Supplementary material for: Exclusive enteral nutrition remodels the intestinal flora in patients with active Crohn's disease
Source: BMC Gastroenterol. 2022 Apr 30;22:212. doi: 10.1186/s12876-022-02293-y (PMC9059691; doi:10.1186/s12876-022-02293-y)
Supplement: Supplementary file 1 — Additional file 1: Table S1. The effective sequencing sequence statistics of the Fecal sample. [file 12876_2022_2293_MOESM1_ESM.docx]

| **Table** **S1** The Effective Sequencing Sequence Statistics of the Fecal Samples | | | | | | | | |
| --- | --- | --- | --- | --- | --- | --- | --- | --- |
| Sample | Raw_Tags | Raw_Bases | Valid_Tags | Valid_Bases | Valid% | Q20% | Q30% | GC% |
| Prior_01 | 86874 | 43.44M | 84547 | 36.08M | 97.32 | 97.69 | 92.98 | 54.13 |
| Prior_02 | 65758 | 32.88M | 54437 | 22.39M | 82.78 | 89.39 | 76.44 | 54.14 |
| Prior_03 | 82157 | 41.08M | 58102 | 23.80M | 70.72 | 94.51 | 85.59 | 52.73 |
| Prior_04 | 86928 | 43.46M | 82633 | 34.31M | 95.06 | 98.28 | 94.72 | 53.70 |
| Prior_05 | 80486 | 40.24M | 77239 | 32.62M | 95.97 | 98.27 | 94.76 | 54.52 |
| Prior_06 | 86231 | 43.12M | 84147 | 35.88M | 97.58 | 98.20 | 94.46 | 52.96 |
| Prior_07 | 82951 | 41.48M | 72988 | 30.97M | 87.99 | 98.53 | 95.29 | 53.32 |
| Post_01 | 85877 | 42.94M | 82576 | 33.54M | 96.16 | 98.58 | 95.54 | 53.70 |
| Post_02 | 86883 | 43.44M | 83522 | 34.90M | 96.13 | 98.35 | 94.90 | 53.74 |
| Post_03 | 86232 | 43.12M | 84329 | 34.47M | 97.79 | 96.36 | 89.93 | 51.98 |
| Post_04 | 83999 | 42.00M | 79096 | 32.37M | 94.16 | 93.62 | 83.52 | 53.54 |
| Post_05 | 57388 | 28.69M | 56231 | 23.38M | 97.98 | 98.48 | 95.23 | 53.99 |
| Post_06 | 85801 | 42.90M | 79164 | 33.14M | 92.26 | 98.62 | 95.59 | 54.94 |
| Post_07 | 84481 | 42.24M | 70312 | 29.06M | 83.23 | 97.15 | 91.71 | 51.29 |
